# Dynamic FDG PET/CT imaging: quantitative assessment, advantages and application in the diagnosis of malignant solid tumors

**DOI:** 10.3389/fonc.2025.1539911

**Published:** 2025-04-14

**Authors:** Yiling Wang, Yuanshan Yang, Jinxin Li, Dezhou Cheng, Hai Xu, Jinbai Huang

**Affiliations:** ^1^ Nuclear Medicine Department, The First Affiliated Hospital of Yangtze University, Jingzhou, Hubei, China; ^2^ Department of Medical Imaging, Health Science Center, Yangtze University, Jingzhou, Hubei, China; ^3^ Division of Endocrinology and Rheumatology, HuangPi People’s Hospital, The Third Affiliated Hospital of Jianghan University, Wuhan, China

**Keywords:** dynamic PET, dynamic acquisition, quantification, oncology, clinical application

## Abstract

Dynamic imaging has obtained remarkable achievements among a variety of malignant tumors due to the development of multiple simplified scanning protocols and the emergence of whole-body PET/CT scanners, which promote wider application of dynamic PET/CT. In this paper, we mainly review the acquisition protocols of dynamic imaging, related kinetic parameters, advantages and the application of dynamic PET/CT imaging in malignant tumors, including lung cancer, hepatocellular carcinoma, breast cancer, pancreatic carcinoma, prostate neoplasm, and cancer of head and neck. Dynamic PET/CT imaging is increasingly being applied the diagnosis, staging, efficacy monitoring, and prognosis evaluation of malignant tumors. Although standardized uptake value is the most frequently employed semi-quantitative assessment index for static imaging, it is susceptible to several factors, thus cannot be used to evaluate the tracer kinetic information of the lesion. Dynamic PET/CT imaging can be used to achieve continuous assessment of the metabolic activity of a lesion over a certain time frame through quantitative measurement of kinetic parameters, such as the net uptake rate constant. Compared with conventional static imaging, dynamic scanning can be used for the early estimation of minute metabolic changes in tumors. Besides, dynamic scanning can directly and effectively reflect tracer uptake. Nevertheless, the intricacy of parameter analysis and the lengthy scanning time related to dynamic scanning limits its clinical application. Dynamic imaging has obtained remarkable achievements among a variety of malignant tumors due to the development of multiple simplified scanning protocols and the emergence of whole-body PET/CT scanners, which promote wider application of dynamic PET/CT. In this paper, we mainly review the acquisition protocols of dynamic imaging, related kinetic parameters, advantages and the application of dynamic PET/CT imaging in malignant tumors, including lung cancer, hepatocellular carcinoma, breast cancer, pancreatic carcinoma, prostate neoplasm, and cancer of head and neck.

## 1 Introduction

Positron emission tomography/computed tomography (PET/CT) is a molecular imaging technique widely used to identify and analyze physiological, pathological, biochemical, and metabolic alterations in human tissues through radiolabeled tracers. PET/CT can be used to diagnose and evaluate various diseases. Although standardized uptake value (SUV) is a widely used parameter to measure the amount of tracer uptake in both healthy and diseased tissues, it is influenced by several factors, such as physiological factors (body weight, blood glucose levels, and respiratory movement of the patient), procedural factors (injection dose, imaging time, and region of interest (ROI) outlining), and physical factors (partial volume effect, acquisition mode) (1). The degree of glucose metabolism in tumors correlates with factors such as histological type, tumor grade and size. For instance, the SUV_max_ of squamous carcinoma, poorly differentiated adenocarcinoma, small cell carcinoma and large cell carcinoma was significantly increased, while the SUV_max_ of well-differentiated adenocarcinoma, carcinoma *in situ* and minimally invasive carcinoma was only mildly increased or not significantly increased. Even for lung cancers of the same histologic type, SUV_max_ was only mildly increased in low-grade and significantly increased in high-grade. In addition, due to the influence of spatial resolution, the increase in SUV of lung cancer <1 cm is not obvious (2, 3). Furthermore, the problem of false positives is still widespread, and active infectious lesions such as pneumonia and tuberculosis can be characterized by a marked increase in SUV_max_ (3). This is due to the fact that ^18^F-FDG is not a tumor-specific imaging agent, and the SUV value is the cumulative activity value of ^18^F-FDG (4), therefore, SUV is unable to accurately distinguish between tumors, inflammation and normal physiological metabolism.

Dynamic PET (dPET) can reflect the physiological and metabolic information of the tumor from the time of the injection of the imaging agent. This is achieved by extracting kinetic parameters through kinetic modeling, including the uptake rate constant K_i_, the metabolic rate of FDG (MR_FDG_), the time activity curves (TACs), and other parameters. Quantitative assessment of the images can be achieved using dPET by avoiding the influence of various factors, such as body mass index and injection-imaging time. Nonetheless, some factors such as excessive noise, the requirement for arterial blood collection as an input function (5), and long scanning times limit the clinical application of dPET. Conventional PET scanners have a limited axial of view (aFOV) (about 25 cm), restricting dPET imaging of a single organ away from the blood pool, such as the lower abdomen, pelvis, and extremities. The recently established long-axis field-of-view whole-body PET systems have enhanced signal-to-noise ratios by utilizing 3D acquisition modes, high-efficiency bismuth germanate detector crystals, and lengthening the length of the system in the axial direction to capture more detection counts. Additionally, the advanced PET systems enhance spatial resolution by transitioning from large conventional photomultiplier tubes to compact solid-state silicon photomultipliers. The optimization of time-of-flight techniques and image reconstruction techniques, such as point diffusion, enhances the precise positioning of the annihilation point on the line of response, further improving the temporal resolution and reducing the image noise. These developments may facilitate pharmacokinetic studies and provide a foundation for the application of novel imaging agents to improve the acceptance of dynamic imaging in the clinical setting. This review aimed to describe the acquisition modalities of dPET and the relevant kinetic parameters, discuss the advantages of dynamic imaging, and provide an overview of the application of dynamic imaging in malignant tumors.

## 2 Quantitative assessment of dynamic PET

### 2.1 dPET data acquisition

dPET acquisition captures the spatial distribution of the captured radiotracer changes over time. The dynamic scanning in the aFOV of conventional PET/CT 15 to 25 cm is mainly performed in two ways: step-and-shoot scan mode (SS) and continuous bed motion (CBM). In SS, the number of beds is fixed, and scans must be selected to fit within these beds, with adjacent beds having overlapping sections that can cause partial volume effects. In contrast, CBM does not have fixed beds, allowing for customizable scanning ranges. The absence of bed overlap reduces artifacts and enhances patient comfort, leading to shorter acquisition times and improved efficiency (6).

**Figure 1:**
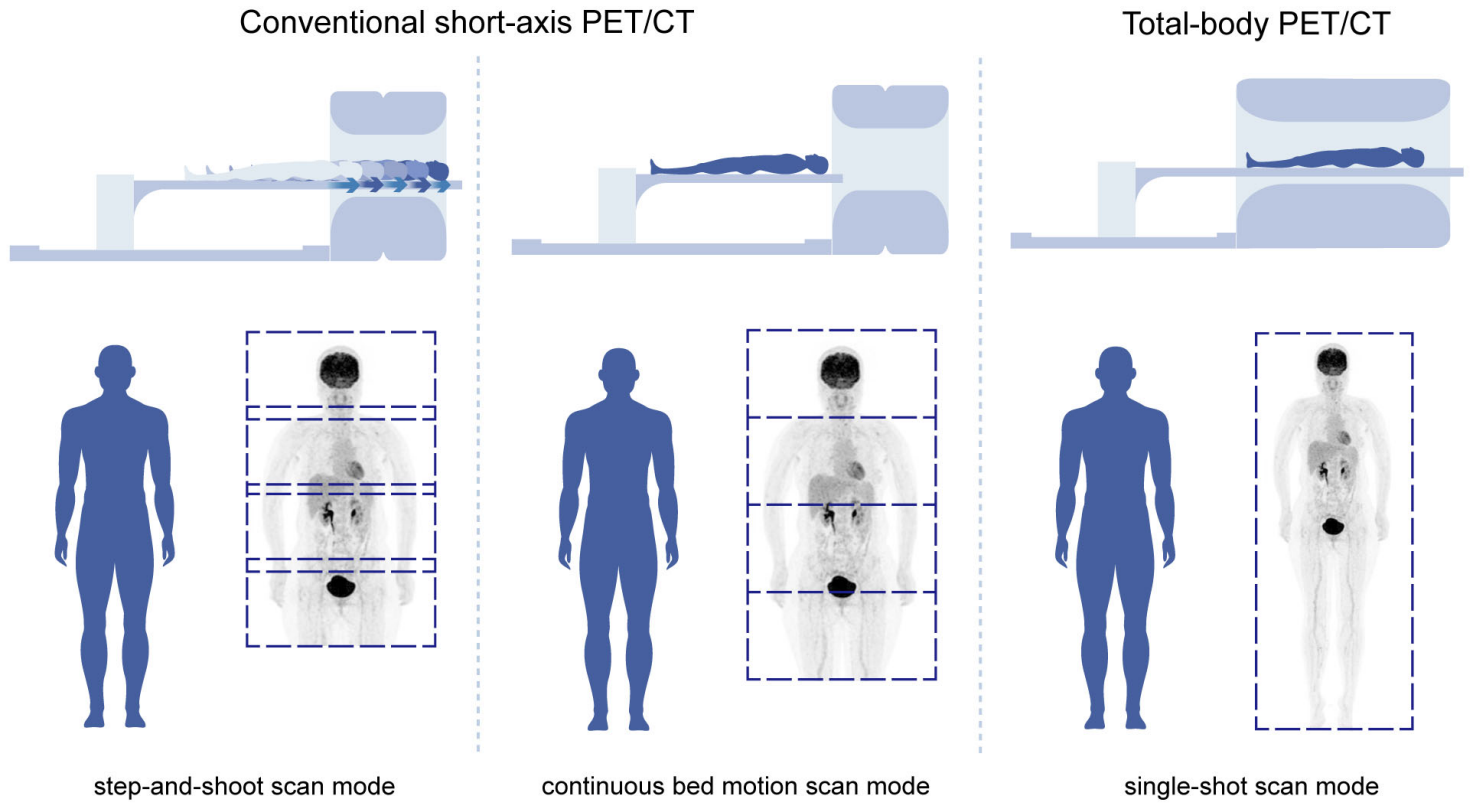
Comparison of conventional short-axis PET/CT and total-body PET/CT.

Conventional PET scanners cannot concurrently capture dynamic data at the far end of the body because of the limits of the short-axis field of view. Therefore, increased axial coverage in a whole-body PET scanner may achieve kinetic analysis on lesions outside of the traditional aFOV. This eliminates time gaps and improves sensitivity, thus eliminating the need for arterial blood collection, which can lead to smaller doses of medication. A new generation of PET scanners entered the market in late 2018, leveraging the most recent advancements in electronics and materials research to attain scanning fields of 1 to 2 meters. These scanners can complete a whole-body acquisition in less than 60 seconds, providing real-time insight into pathophysiologic processes (**Figure 1**).

### 2.2 Relevant kinetic parameters of dPET

**Figure 2:**
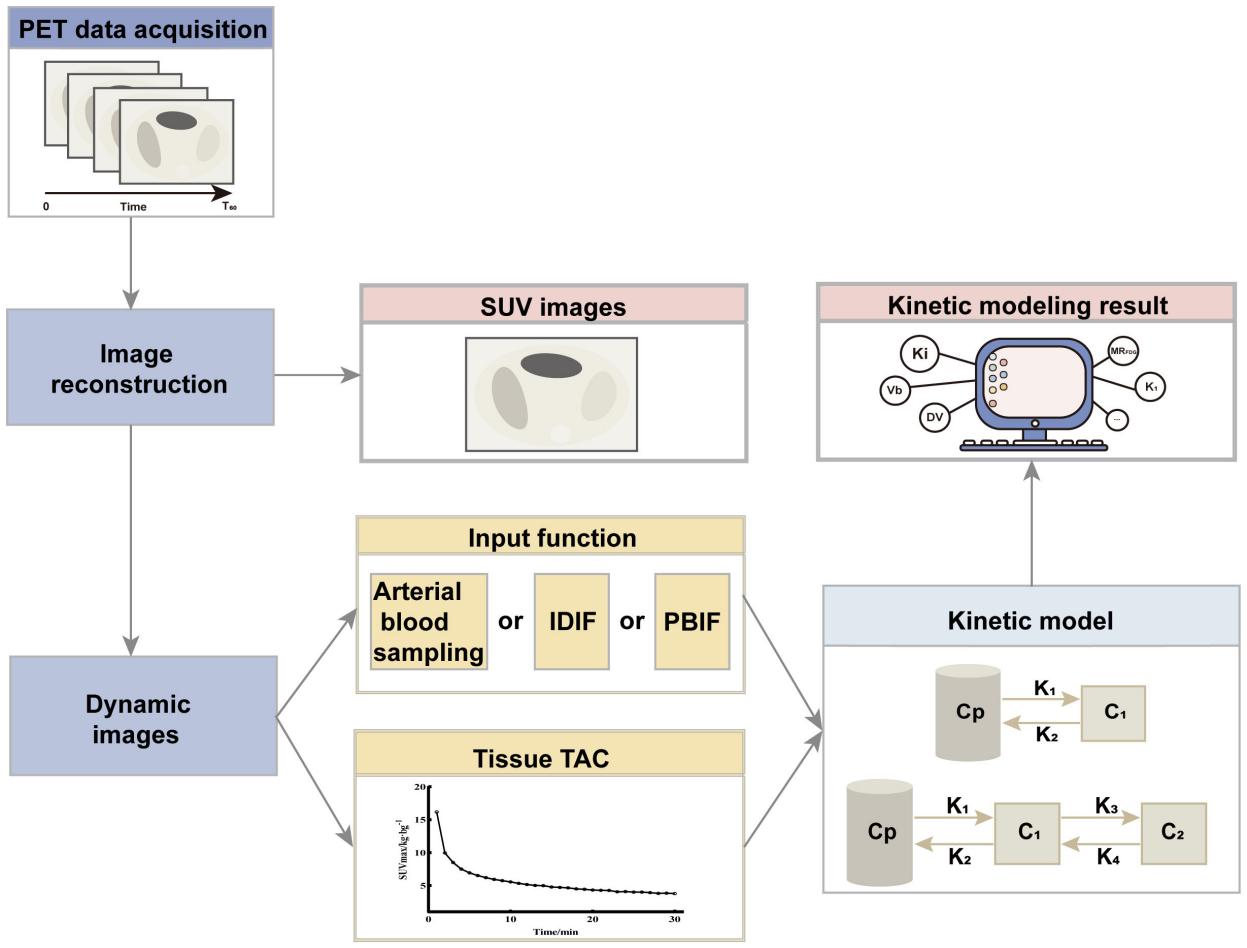
Flowchart of PET kinetic modeling and parametric imaging. PET images are acquired over a period of time (generally 0-60 min), and the 60th min data are reconstructed to obtain SUV images. Further all the data are reconstructed to get the dynamic image. For each image voxel or region of interest, the TAC is extracted from the dynamic sequence, and kinetic parameters (K_i_, V_b_, and MR_FDG_, etc.) are estimated using kinetic models and input functions. The input function can be measured invasively by arterial blood sampling, derived non-invasively from the dynamic images (Image-derived input function, IDIF) or a population-based input function (PBIF). Evaluation of kinetic modeling can be based on a two-tissue compartment model or a three-tissue compartment model. C_p_ is the plasma compartment; C_1_ and C_2_ are tissue compartments.

SUV is the most commonly used quantitative indicator of radiopharmaceutical uptake. SUV is a simple semi-quantitative analytical technique obtained by sketching the concentration of activity in a lesion after a period of rest from injected radiopharmaceuticals. K_i_ images and DV images that have been kinetically modeled have better contrast compared to SUV images. Compartmental models are frequently used in the clinic for the evaluation of kinetic models, and the most commonly used compartmental models in PET are the two-tissue compartment model and the three-tissue compartment model, which describes the exchange of radiopharmaceuticals between the blood and tissue compartments, where the four transport rates are represented by the meanings of K_1_ related to the inflow of tracer from the blood compartment into the tissue compartments, K_2_ related to the efflux, K_3_ reflecting the rate of phosphorylation, and K_4_ reflects the rate of dephosphorylation. Whereas the parameter that allows the estimation of partial blood volume, also known as vascular density (V_b_). V_b_ and K_1_ are correlated and are usually higher than the rate of phosphorylation(K_3_).This model differs from the model that does not consider K_4_ and V_b_, as proposed by Sokoloff et al. The absence of K_4_ and V_b_ results in different values of K_1_ and K_3_, as K_1_ is dependent on V_b_, whereas K_3_ is dependent on K_4_. In addition, the dephosphorylation rate (K_4_) of ^18^F-FDG may be low but cannot be ignored. Compartmental models have the advantage of directly visualizing different kinetic parameters, but it is computationally intensive and sensitive to noise. As an alternative to compartmental modeling, the Patlak model is a linear graphical method that can approximate K_i_ using the slope of the graphical curves of the blood input function and the tissue time-activity curve (TAC), and obtains intercepted images related to the volume fraction and the volume of distribution. For parametric imaging, the Patlak model has the advantages of computational efficiency and noise robustness. The associated PET kinetic modeling and parametric imaging flow is shown in **Figure 2**.

#### 2.2.1 Ki

K_i_ represents the uptake rate of radiopharmaceuticals into tissues and is a quantitative measure of metabolism from the Patlak slope. K_i_ can be determined using dynamic imaging to acquire the TAC and artery input function (AIF). Although SUV and K_i_ are not similar, they exhibit a correlation. SUV measures total activity (both metabolic and nonmetabolic) in a lesion, whereas Patlak analysis separates metabolic and non-metabolic activity and determines only metabolic uptake. Therefore, K_i_ is considered to be a more accurate indicator of glucose metabolic rate than SUV. Besides, K_i_ has a greater level of sensitivity than SUV images and thus has better sensitivity (from 92.5% to 95%) and accuracy (from 90.24% to 95.12%) in diagnosing malignant tumors (7) and potentially enhanced specificity. Fotis Kotasidis et al. conducted multichannel whole-body ^18^F-FDG PET parametric imaging in 8 patients with staged lung and liver lesions. They found that three malignant liver lesions, confirmed by contrast-enhanced CT, and one biopsy-confirmed hepatic malignant lesion, were not visible on static SUV or Patlak images. However, these lesions were clearly identified on K_1_ and K_2_ images, and the K_i_ images enhanced lesion detectability (8). Oncology Patlak K_i_ imaging complements standard SUV imaging to achieve equal or higher lesion detection rates, especially in tissue organs with a rich blood supply (e.g., liver), where K_i_ images significantly reduce background uptake, resulting in an increased tumor-to- background ratio(TBR), which makes it easier to detect lesions (9). Moreover, due to the higher TBR, K_i_ images are more accurate in outlining the radiotherapy target area and volume of interest. K_i_ images have similar (or lower) quality compared to SUV images. Also, significant motion artifacts may be apparent in K_i_ images during prolonged dynamic scanning.

In addition, the time it takes for tracers to reach different organs and lesions can vary significantly. Long axial field-of-view PET/CT, which does not require continuous bed movement, allows for precise capture of tracer pharmacokinetics in major organs and most lesions, offering high temporal resolution (10). Christos Sachpekidis et al. (11) used a two-tissue model for 38 cases of prostate cancer patients for whole-body dynamic imaging and found significantly higher parameter K_3_ values in prostate lesions and parotid glands than in the liver and spleen, reflecting higher tracer binding and internalization rates in diseased tissues. Further, rapid temporal sampling provides the opportunity to capture blood volume or blood flow (perfusion), which are potential biomarkers for predicting treatment response or survival. Data indicates that the mid-term dynamic ^18^F-FDG PET/CT Patlak parameter K_i_max can predict the prognosis of patients with diffuse large B-cell lymphoma and that predictive modeling by K_i_max and mid-term response to treatment permits accurate stratification of prognostic risk in diffuse large B-cell lymphoma (12).

#### 2.2.2 MR_FDG_ and DV_FDG_

MR_FDG_ and Distribution volume of free FDG(DV_FDG_) are quantitative metrics that describe parametric images. MR_FDG_ and DV_FDG_ are calculated by fitting a linear regression function to the time-activity data and each pixel based on dPET data. The slope of the image indicates the metabolic rate of FDG entering the tissue based on the contrasting effect on the surrounding tissue. This information can be utilized to identify malignant lesions and determine the placement of volumes of interests (VOIs). The intercept of the image represents DV_FDG_, which can be used for better anatomical localization of the lesion. André H Dias et al. performed dynamic whole-body PET/CT scans of 103 patients using long axial field-of-view PET/CT and compared static whole-body ^18^F-FDG PET/CT with multiparametric Patlak images and found that MR_FDG_ images can reduce false-positive rate, identify false-positive lesions misdiagnosed by the SUV images. They also showed that MR_FDG_ images provide a higher TBR and contrast-to-noise ratio(CNR) (13). Similarly, Pedersen et al. (14) performed whole-body dynamic imaging of patients with locally advanced breast cancer and found that MR_FDG_ images had better lesion visibility and 2.28 times higher TBR than SUV images, which effectively improved the detection rate of locally advanced breast cancer lesions. DV_FDG_ images can identify small SUV artifact foci and improve the specificity of PET/CT. DV_FDG_ images reflect free FDG, whereas MR_FDG_ images reflect metabolic FDG. SUV images are equivalent to the “sum” of MR_FDG_ and DV_FDG_ images. Therefore, artifactual foci are usually not detected on MR_FDG_ images but can be detected on DV_FDG_ images and SUV images. In terms of benign and malignant differentiation, MR_FDG_ and DV_FDG_ may be robust indicators for identifying benign and malignant lung nodules. It has been demonstrated that most malignant lung nodules exhibit high uptake on MR_FDG_ and SUV, while benign lesions exhibit low uptake on MR_FDG_ but high uptake on DV_FDG_ (15). Moreover, factors such as different pathological types, lesion sizes and methods of lesion delineation can influence MR_FDG_ results (16). In a direct comparison of whole-body PET scans from 45 patients, Wu et al. (17) found that the improved image quality of MR_FDG_ did not necessarily result in significant differences between benign and malignant lesions. This may be because MR_FDG_ is particularly useful for lesions surrounded by high background activity, but it provides limited additional information for lesions with low uptake and low background activity.

#### 2.2.3 FD

Fractal dimension (FD) is used to measure the heterogeneity of the VOI in the non-compartmental model- FD can be determined by analyzing the time-activity data of each individual voxel within the VOI. The FD number ranges from 0 to 2, suggesting either a deterministic or chaotic distribution of tracer activity. A higher FD value indicates a more varied and uneven distribution of the tracer activity (18). Therefore, the FD value reflects the degree of benignity or malignancy of the tumor(malignant lesions have higher FD values than benign lesions). However, parametric images do not offer substantial additional insights into tumor heterogeneity when compared to static images. Meijer et al. delineated lesion volumes on both static and parametric images in 35 non-small cell lung cancer (NSCLC) cases for radiation treatment planning. They subsequently compared the volume measurements obtained from PET images with pathological volumes, as well as the volumes associated with different NSCLC tissue subtypes. The results showed that the pathological volume was larger than the PET volume, the glucose metabolism rate, ^18^F-FDG phosphorylation rate and heterogeneity of VB were lower in the adenocarcinoma group than in the squamous carcinoma group, and there was no difference in the parametric images and static images of NSCLC (19). Similarly, Tixier et al (20) extracted heterogeneity parameters from static and parametric images of 20 NSCLC patients and showed that the differences between static and parametric images in quantitative measures of intra-tumor heterogeneity were mainly attributed to variations in image features and noise, rather than to substantial differences in the spatial distribution of uptake within the actual tumor.

## 3 Advantages of dPET

### 3.1 Use of non-invasive input functions

Kinetic modeling analysis of ^18^F-FDG using PET/CT requires AIF based on TAC image-derived AIF. Although arterial blood collection is the gold standard for obtaining AIF (21, 22), it is not the clinically preferred method due to its invasive nature during the procedure (23). Image-derived input function (IDIF) is currently the most commonly used input function. IDIF allows the selection of a dynamic scan of the initial blood pool (heart or aorta) in place of arterial blood collection. The ascending aorta is widely chosen to obtain IDIF because of the strong correlation between the results of the ascending aorta and arterial sampling. Besides, a relatively large ROI can be determined when using an ascending aorta with better statistical properties and less interference from adjacent myocardial spillovers. However, the accuracy of using ascending aorta is affected by body motion and partial volume effects (23). Zhu et al. used IDIF from small vascular regions (carotid artery) as a merging kernel of *a priori* information, stabilizing the performance of the simultaneous estimation of the input function method and significantly enhancing the accuracy of evaluating early time points of the input function (24). Besides IDIF, a template can be generated using a population-based input function (PBIF). PBIF can be obtained by averaging the arterial blood data collected from the subjects, predetermining the model parameters, and applying a scaling factor for normalization for each subject. Mika et al. compared PBIFs normalized by different scaling factors and found good accuracy and precision using a scaling factor consisting of the area under the curve of the time window and the initial volume of distribution of the developer (22). However, using a single model with a single input function from a single blood pool can lead to inaccurate kinetic parameter estimates for organs with dual blood supplies (liver and lungs) (25). Wang et al. proposed that dynamics can be modeled using an optimally derived dual-blood input function (DBIF). Compared with the traditional single-blood input function (SBIF) and PBIF, DBIF can significantly improve the fitting of liver time-activity curves without the need to invade the arteries for blood sampling or delineate additional regions of interest. Besides, the optimized model demonstrated that FDG blood-hepatic transit rate K_i_ is significantly correlated with the histological grading of liver inflammation, making it suitable for individual patients (26). Similarly, Yiran Wang et al. used high-temporal resolution dynamic imaging and demonstrated that the DBIF model can be used to better quantify the kinetic parameters and improve the quality of the fit of the efficiency curves under the influence of dual blood supply to the lungs. They also showed that this effect is particularly pronounced in lung tumors (27). In addition, no input function is required in the non-compartmental model to reflect the changes in tumor heterogeneity. The temporal activity data of the tracer in a single voxel within the ROI can be evaluated to show the non-homogeneity of the distribution of the tracer activity over time within the ROI, which is closely correlated with the pathological findings (5, 28, 29). It is also possible to acquire image IDIFs from multiple blood pools (e.g., ventricles and arteries) simultaneously by expanding the axial scanning field of view, such as with long axial PET/CT, and selecting the most relevant vascular IDIFs for kinetic modeling (30).

### 3.2 Short-term dynamic acquisition

**Figure 3:**
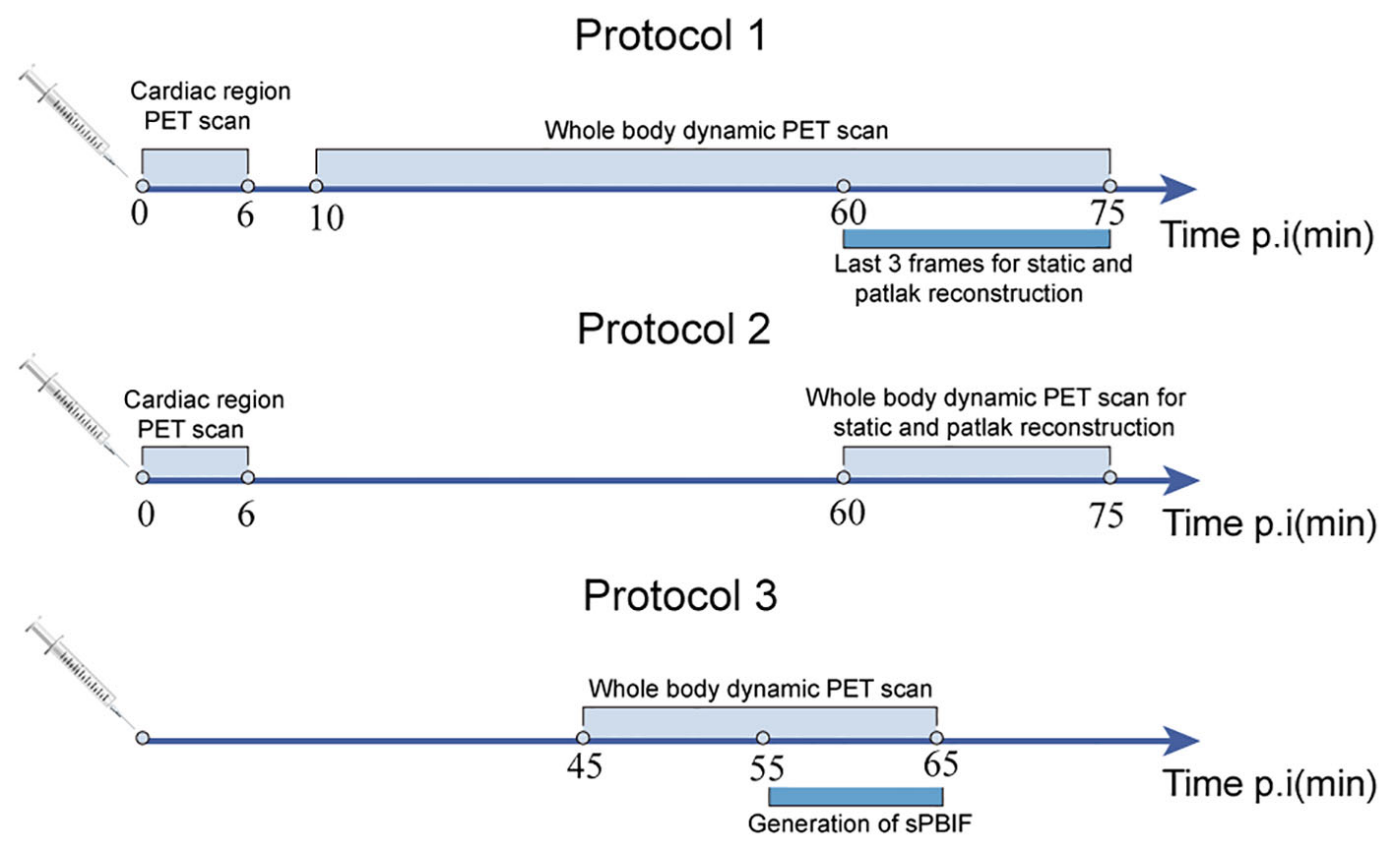
Three different imaging schemes for shortened scanning protocols: standard 75 min dynamic scanning (protocol 1), dual time-point imaging (protocol 2), and 20 min dynamic scanning based on population input function (protocol 3).

Although dynamic scanning allows continuous tracking of changes in tracer activity, it is associated with long scanning times, poor patient tolerance, and limited clinical application. The ultra-high-sensitivity whole-body PET scanners can shorten the time for dynamic acquisition. Feng et al. demonstrated that ultra-high sensitivity whole-body PET scanner can achieve whole-body parametric image reconstruction at an early stage of scanning (within 2 minutes of injection) (31). Wu et al. also demonstrated that a nonlinear estimation method can reduce the acquisition time of FDG PET K_i_ parametric imaging (32). However, Wu et al. also indicated that the shorter imaging time (10 min) is associated with a significantly increased computational cost. Several researchers have used simplified dynamic scanning protocols to effectively shorten the dynamic acquisition time. Compared with the 0-75 min standard parametric images, Hui Wang et al. found that using two short-duration dynamic multiparametric images of 0-6 min (input function) and 60-75 min (equilibrium activity) generated from 0-75 min standard parametric images provide good image quality. Dual time-point imaging can effectively improve gastrointestinal motility artifacts in standard parametric images due to long scanning times by leaving the scanner in the middle of the scanning process (33). Meanwhile, this needs the use of regions of interest from both aortas in both scans to generate the full IDIF and two CT scans to attenuate the two PET images for correction, increasing the radiation dose. Alternatively, PBIF can be used to scale the IDIF extracted from the later time frames of the scan, and a scaling curve can be used to generate the Patlak K_i_ values. However, the shape of the input curve must be assumed to be the same for each patient. Hasan Sari et al. explored the effect of different scanning cycles on the accuracy of sPBIF and found that sPBIF generated from 55-65 min of image data can accurately assess K_i_ (deviation <5%) and provide good image quality when it is used for K_i_ images generated from 20 min (45-65 min post-injection) dynamic scanning (34). Alternatively, a global scaling factor can be used to normalize the Patlak slopes obtained from the data in the later frames, thus eliminating the need for PBIF (35). Although some potential inaccuracies may occur, images obtained using post-scanning give better target-to-background ratios than static images, effectively reducing scanning time (**Figure 3**).

### 3.3 High sensitivity

Whole-body dynamic scanning is mainly achieved by SS and CBM scanning modes in conventional PET scanners due to the limitations of the detector axial scanning field. The high sensitivity of the continuous bed motion listing mode is mainly reflected in the provision of continuous short-interval whole-body dynamic scanning. The whole-body dynamic images are acquired by multi-channel and multibed PET acquisition. Motoki Tomohito Kaji et al. found that the use of CBM scanning mode, which removes the frames with motion in the images, can improve the quality of image acquisition for patients who may move during the scanning process (36). In addition, it is sometimes difficult to identify lesions in the urinary tract and gastrointestinal tract using conventional static PET imaging. As a result, radiologic technicians are needed to distinguish whether the gastrointestinal tract is pathologically ingested or physiologically ingested through delayed imaging. Besides, Motoki Nishimura et al. found that continuous dynamic whole-body imaging can differentiate between pathologic and physiologic uptake based on changes in uptake shape, minimizing the need for such delayed imaging and reducing radiation dose (36, 37). Although multibed, multichannel approaches can provide dynamic imaging with extended AFOV, their temporal resolution is limited by bed motion and scanner sensitivity, which cannot rapidly capture tracer kinetics. In contrast, long-axis field-of-view PET/CT permits dynamic imaging of the entire body at high temporal resolution, acquisitions are no longer segmented by bed position. The long-axis field-of-view PET/CT eliminates the need for segmenting bed position during acquisitions. Besides, long-axis field-of-view PET/CT significantly improves sensitivity by increasing the number of detectors, allowing for dynamic whole-body acquisitions and improved image quality, increasing the detectability of microscopic lesions. Guillaume Fahrni et al. compared SUV and K_i_ images for 18 different oncologic indications (lesion characterization and staging) and found that K_i_ can improve sensitivity (from 92.5% to 95%) and accuracy (from 90.24% to 95.12%) compared with SUV imaging, especially for organs with high background uptake (liver). This is because K_i_ images suppress the non-specific ^18^F-FDG signal in the background, resulting in higher contrast in the region of abnormal uptake, improved lesion detectability, and reduced rate of false positives of lesions (7). In addition, the effect of respiratory motion in routine PET scans may lead to inaccurate description (tumor size, location, and morphology) and quantification of small lung nodules near the diaphragm. The use of breath-holding PET technology combined with the counting performance in the long aFOV can minimize motion artifacts and PET/CT image mismatches, especially for small nodules ≤10 mm from the pleura, effectively reducing the false-negative rate of early lung adenocarcinoma (38).

## 4 Application of dPET in the diagnosis of malignant solid tumors

**Figure 4:**
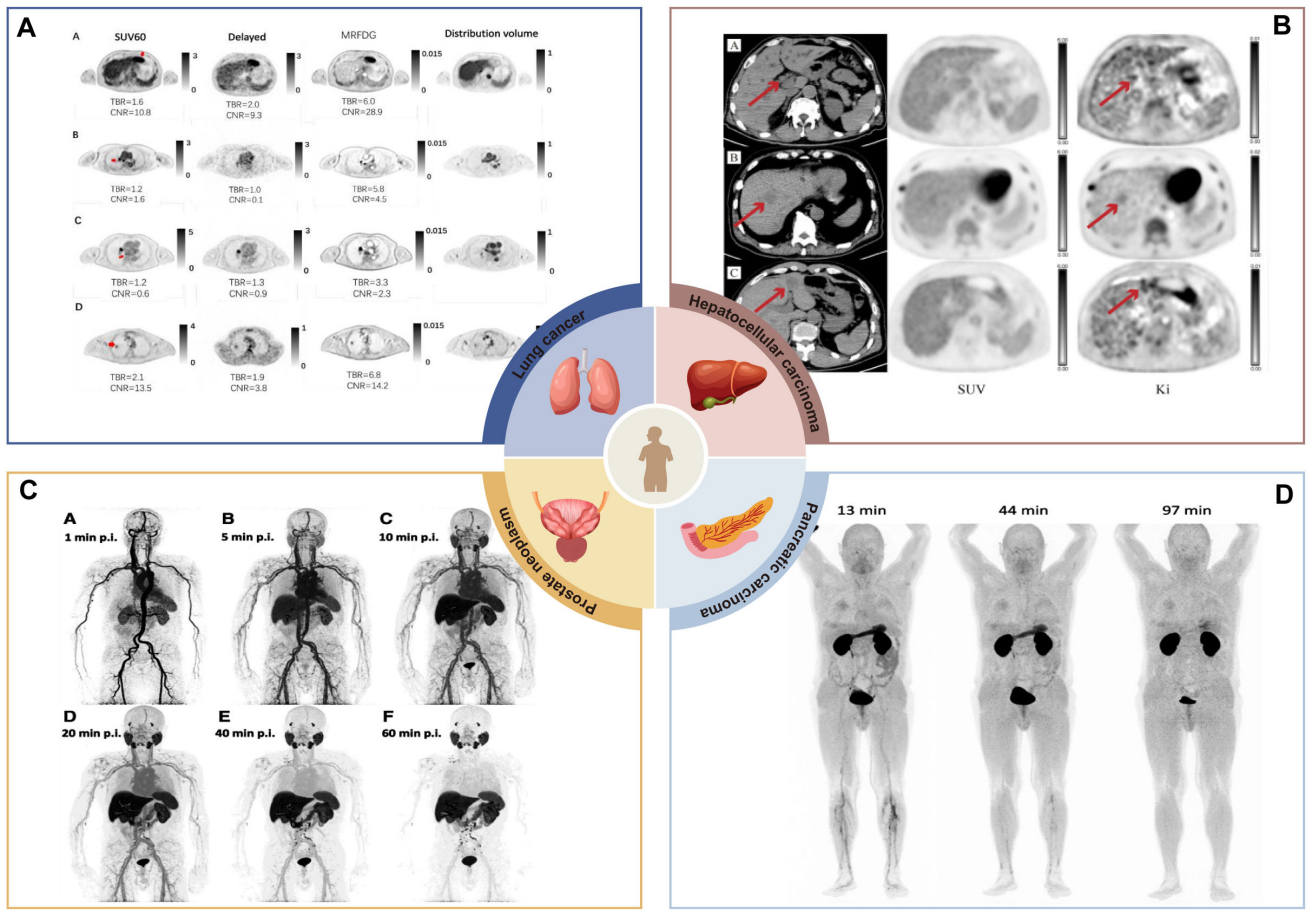
Clinical application of dPET in partial solid tumor studies. **(A)** MR_FDG_ images improve the recognition of suspicious primary lesions. Primary suspicious lesions of hepatocellular carcinoma, mediastinal lesions, and lung cancer cannot be accurately identified in conventional static images, but they can be clearly distinguished in MR_FDG_ images (17). Image taken from Yaping Wu et al. CC BY 4.0 License: http://creativecommons.org/licenses/by/4.0/. **(B)** K_1_ images were used to show positive lesions masked by physiologic background uptake. A case of hepatocellular carcinoma, the lesion was not metabolized in SUV images but was detected on K_i_ images (9). Image taken from Christos Sachpekidis et al. CC BY 4.0License: http://creativecommons.org/licenses/by/4.0/. **(C)** Dynamic PET was applied to study the systemic pharmacokinetics of ^18^F-PSMA-1007 in a group of prostate cancer patients. The figure shows dynamic PET acquisitions of a patient with PC biochemical recurrence at various time points. The patient had multiple iliac, retroperitoneal and supraclavicular manifestations of high uptake lymph node metastases. Of note, some retroperitoneal lymph node metastases were visualized within 10 minutes of tracer injection (11). Image taken from Christos Sachpekidis et al. CC BY 4.0License: http://creativecommons.org/licenses/by/4.0/. **(D)** Dynamic imaging was applied to estimate organ distribution and dosimetry data for a new contrast agent (^68^Ga-Trivehexin). In addition to the kidneys and bladder, there was a significant uptake in the stomach, which disappeared 1.5 hours after injection (74). Image taken from Neil Gerard Quigley et al. CC BY 4.0 License: http://creativecommons.org/licenses/by/4.0/.

Dynamic imaging has had a significant impact on the diagnosis and management of tumor patients and has gained initial use worldwide. This article focuses on malignant solid tumors for which dynamic imaging has been more commonly used in clinical practice and summarizes the characteristics of the relevant studies (**Table 1**). **Figure 4** summarizes the clinical applications of dPET in partial solid tumor studies.

**Table 1:** Summary of the selected PET/CT oncological studies applying dynamic imaging.

Tracer	Tumor Type	No.of patients	Acquisition Program	Software Used	Goal	References
^18^F-FDG	benign and malignant pulmonary lesions	147	dPET/CT (65 min, chest) + sPET/CT (10-20 min, whole body)	Matlab in house software	Impact of parametric Patlak imaging on distinguishing benign and malignant lung lesions and predicting EGFR mutation status	(40)
^18^F-FDG	metastatic and non-metastatic LNs in lung cancer	108	dPET/CT (65 min, chest) + sPET/CT (10-20 min, whole body)	Matlab in house software	Impact of parametric Patlack imaging on the differentiation of metastatic and non-metastatic LNs in lung cancer	(41)
^18^F-FDG	NSCLC	60	dPET/CT (60min, chest) + sPET/CT (10-20 min, whole body)	in house software	Comparison of quantitative and semiquantitative parameters in predicting OS in NSCLC patients scheduled to receive platinum-based chemotherapy	(43)
^18^F-FDG	locally advanced NSCLC	37	dPET/CT (60min, whole body)	in house software	Impact of parametric Patrak imaging on metabolic profiles after induction immunochemotherapy in patients with locally advanced NSCLC	(44)
^18^F-FDG	locally advanced NSCLC	15	dPET/CT (four timepoints, whole body)	in house software	Evaluation of parametric imaging for different radiation treatment plans	(45)
^18^F-FDG	HCC	10	dPET/CT (60 frames of 10s, 50 frames of 60s, liver)	in house software	Comparison of four kinetic models with different dual blood input functions	(46)
^18^F-FDG	HCC,ICC	24	dPET/CT (60min, liver)	in MIM software Inc	Evaluation of a two-tissue compartment model with dual blood input function	(48)
^18^F-FDG	HCC	17	early dPET/CT (5min, liver) + sPET/CT (10-20 min, whole body)	in house software	Evaluating the feasibility of a dual-input dual-compartment uptake model for the diagnosis of HCC by perfusion and early uptake	(50)
^18^F-FDG	HCC	22	early dPET/CT (2min, liver) + sPET/CT (10-20 min, whole body)	in house software	Evaluating the diagnostic value of early dynamic combined conventional imaging for HCC	(49)
^18^F-FDG	BC	34	dPET/CT (60min, chest)	PMOD	Evaluate the correlation between the pathologic features of BC and kinetic parameters	(60)
^18^F-FDG	locally advanced BC	35	dPET/CT (60min, chest) + sPET/CT (10-20 min, whole body)	PMOD	Evaluation of metabolism/perfusion mismatch in breast tumors by dynamic parameters combined with MRI parameters	(61)
^18^F-FDG	locally advanced BC	10	dPET/CT (6min, chest) + dPET/CT (64min, whole body)	PMOD	Comparison of detection rates of locally advanced BC on conventional SUV images and parametric images	(14)
^18^F-FDG	BC	217	Dynamic first-pass(2min, chest) + sPET/CT (two-step, chest)	in house software	Comparison of parametric and SUV images in tumor perfusion and metabolism	(67)
^68^Ga-FAPI	IPMN	25	sPET/CT (10-20 min, whole body) + dPET/CT(45min, whole body)	PMOD	Comparison of the efficacy of static and dynamic imaging in identifying IPMN subtypes	(70)
^68^Ga-PSMA-11	PDAC	49	sPET/CT (10-20 min, whole body) + dPET/CT(60min,pancreas)	in MIM software Inc	Comparison of ^68^Ga-FAPI and ^18^F-FDG in disease staging and prognostic value	(71)
^68^Ga- PSMA-11	prostate cancer	10	dPET/CT (60min, whole body)	PMOD	Evaluation of kinetic and metabolic heterogeneity of tracers in diseased and normal organs	(79)
^68^Ga- PSMA-11	prostate cancer	20	dPET/CT (60min, whole body)	PMOD	Comparison of parametric and SUV imaging in tumor detectability	(81)
^68^Ga- PSMA	prostate cancer	11	dPET/CT (60min, whole body)	in house software	Determining the optimal imaging time for tracers	(91)
68Ga- PSMA-11	prostate cancer	10	dPET/CT (75min, skull vertex to mid-thigh)	PMOD	Evaluation of the possibility of shortening time intervals by investigating dynamic changes in image quality metrics	(82)
^18^F-DCFPyL, ^18^F-FCH PET	prostate cancer	19,23	dPET/CT (22min, prostate and common iliac arteries)	Matlab in house software	Comparison of different kinetic behaviors of two tracers used to interpret different SUV images	(84)
^18^F-FMISO	head and neck cancer	120	dPET/CT (30min, head and neck) + sPET/CT (10 min, head and neck) + sPET/CT (10 min, head and neck)	in house software	Impact of multiparametric imaging on tumor hypoxia, perfusion, and heterogeneity	(87)

NSCLC, non-small cell lung cancer; dPET/CT, dynamic PET/CT; sPET/CT, static PET/CT; EGFR, epidermal growth factor receptor; OS, overall survival; HCC, hepatocellular carcinoma; ICC, intrahepatic cholangiocarcinoma; BC, breast cancer; IPMN, intraductal papillary mucinous neoplasia; PDAC, pancreatic ductal adenocarcinoma.

### 4.1 Lung cancer

Lung cancer is the leading cause of morbidity and mortality worldwide, according to data released by CA in 2024 (39). Therefore, early detection, accurate diagnosis, and development of individualized treatment plans can improve the survival of lung cancer patients. The dynamic metabolic parameter K_i_ can be used to improve specificity when differentiating benign and malignant lung nodules. Studies have shown that the K_i_ parameter is lower in benignancies than in malignancies. Besides, the diagnostic specificity of K_i_ can be further improved (about 0.830) when the critical value of K_i_ is 0.0250 ml/g/min, compared with that of SUV_max_ (40), effectively reducing unnecessary invasive treatment. The K_i_ value is more sensitive than conventional SUV in identifying early lymph node metastasis. Besides, the use of the K_i_ and K_i_/K_1_ values has higher diagnostic specificity in cases where conventional static PET may not accurately identify suspicious lymph nodes (lymph nodes located in the hilar and mediastinal regions), complementing the lower specificity of SUV_max_ (41).

In addition, dynamic imaging is a valuable non-invasive screening method that can be used to assess treatment effectiveness and predict prognosis. Furthermore, SUV and K_i_ values are significantly higher in squamous carcinomas than in adenocarcinomas, possibly due to the differentiation of tumor cells combined with GULT-1 and GULT-3 overexpression (42). K_i_ values are lower in EGFR-expressing adenocarcinoma patients than in patients not expressing EGFR. Also, K_i_ can improve the differentiation in some NSCLC patients who did not undergo EGFR testing (40). Besides K_i_, the tumor load parameters MTV and TLG are reliable overall survival prognostic markers for NSCLC patients receiving platinum-based chemotherapy (43). Notably, the efficacy of a treatment in adjuvant trials is not determined until several years after determining disease-free survival and overall survival. Neoadjuvant trials allow for efficacy endpoints, such as clinical and pathologic response, to be determined in months. Studies have shown that neoadjuvant chemoimmunotherapy can improve long-term survival and increase the chances of cure in patients with unresectable NSCLC. However, there are no suitable predictive imaging markers that can be used to comprehensively characterize the response to neoadjuvant treatment. DaQuan Wang et al. recently demonstrated that the uEXPLORER system can be used for whole-body ^18^F-FDG PET/CT dynamic imaging of patients with locally-advanced NSCLC. The results found that Patlak-K_i_ values were associated with response to induction immunochemotherapy in unresectable locally-advanced NSCLC. Furthermore, they showed that higher FDG K_i_ values have a better response to induction therapy and a higher level of immune cell infiltration in the primary tumor (44). DaQuan Wang et al. further performed repetitive dynamic scanning of patients during combined immuno-radiotherapy and found that the metabolic profile of the high FDG K_i_ group after induction immunochemotherapy was significantly reduced during the treatment period. They also showed that the K_i_ value of the primary foci was significantly correlated with the efficacy and survival of combined immuno-radiotherapy in locally advanced NSCLC patients (45). In summary, dynamic imaging provides non-invasive biomarkers that can predict treatment response in patients with unresectable locally advanced NSCLC.

### 4.2 Hepatocellular carcinoma

Hepatocellular carcinoma (HCC) is one of the leading causes of cancer deaths worldwide. Dynamic imaging, dynamic perfusion imaging, and novel imaging agents have emerged as complementary techniques for HCC diagnosis. The blood input function is crucial for precise analysis in dPET kinetic modeling. Early studies used SBIF from the hepatic artery or portal vein for hepatic kinetic modeling (46). However, recent studies have shown that using DBIF is reasonable because of the dual blood supply to liver tissue from the hepatic artery and portal vein (47). Notably, only a few studies have reported the use of DBIF, and no perfect kinetic modeling has been developed yet. Studies have shown that a reversible dual-tissue compartment model for kinetic modeling using DBIF and optimally derived blood-supply fraction hepatic artery can effectively differentiate malignant lesions from healthy liver tissues. Moreover, the model can be used to further distinguish between lesions of intrahepatic cholangiocarcinoma and HCC (48). Dynamic perfusion imaging focuses on the liver and reveals information about the spatial distribution and temporal dynamics of the tumor through dynamic blood flow parameters, such as time to peak (TTP), blood flow, and hepatic perfusion index starting from the injection of ^18^F-FDG. Therefore, dynamic perfusion imaging enables quantitative interpretation of PET data (49). Studies have shown that TTP and perfusion index can be used to distinguish HCC from background liver tissue, significantly improving the detection rate of poorly differentiated and moderately differentiated HCC (49, 50).

Contrast-enhanced CT or magnetic resonance imaging can also be utilized for HCC screening. The two imaging are associated with many advantages, including multiphase enhancement characteristics and straightforward data gathering. However, the accuracy of these methods may be compromised when the lesion size is less than 2 cm (51). Also, the sensitivity of ^18^F-FDG in detecting HCC is limited to about 36%-68% (52). This is mainly because increased glucose transporter protein expression and hexokinase activity are found in highly differentiated HCC, reducing FDG accumulation in the lesions (53). Some scholars also found that ^68^Ga-FAPI-04 has a higher sensitivity in detecting intrahepatic HCC lesions than ^18^F-FDG, especially for tumors with a diameter of less than 2 cm(diagnostic efficiency: about 68.8%) (54). In addition, ^68^Ga-FAPI-04 showed high sensitivity in identifying liver malignant tumors and metastatic lymph nodes (55, 56).

### 4.3 Breast cancer

Breast cancer (BC) has become the most common cancer in women, surpassing lung cancer, with an estimated 2.3 million new cases reported worldwide each year, and is the leading cause of cancer deaths in women (57). It is classified into four subtypes, including Luminal A, Luminal B, HER-2 overexpression, and triple-negative, based on the expression of cell proliferation markers (K_i_-67), the estrogen (ER), progesterone (PR), and the HER-2 receptor (58). Lack of ER and PR expression in BC correlates with poor overall prognosis, whereas positivity for human epidermal growth factor receptor 2c-erbB2 and K_i_-67 are considered good indicators of the invasiveness and metastastic potential of tumors (59). Kornélia Kajáry et al. conducted dynamic PET/CT imaging on 35 primary breast cancers. Their findings revealed that highly aggressive tumors, characterized by high differentiation, hormone receptor negativity (ER and/or PR), and rapid proliferation (high K_i_-67), exhibited increased cellular uptake and phosphorylation of FDG (60), and these kinetic parameters can be used to characterize residual lesions following neoadjuvant therapy in locally advanced BC because they less dependent on pre-treatment FDG uptake as static parameters. Mid-treatment evaluation of changes in glucose delivery (K_1_) and metabolic rate (MR_FDG_) during neoadjuvant chemotherapy can predict recurrence-free survival (RFS). Specifically, a greater reduction in metabolism-to-perfusion ratios (MR_FDG_/peak PE and MR_FDG_/peak SER) compared to pretreatment levels is associated with longer RFS (61), suggesting a potential new biomarker for therapeutic response in breast cancer. Axillary lymph node metastasis is a critical prognostic factor in locally advanced breast cancer. Traditionally, lymph node status has been assessed invasively through sentinel lymph node biopsy and axillary lymph node dissection (62). These procedures often lead to edema, numbness, pain, and long-term complications (63). The study demonstrated that the TBR and CNR of MR parametric images generated using 6 min single bed dynamic scan and 64 min dynamic whole body PET scan were superior to SUV images, which significantly improved lesion detectability (14).

Microvessel density and tumor angiogenesis can influence the tumor growth and metastasis (64, 65). In contrast, patients with locally advanced breast cancer often exhibit a mismatch between blood supply and metabolic rate within the tumor. This imbalance, potentially caused by inadequate blood flow and/or elevated tumor metabolism, can create a hypoxic microenvironment linked to treatment resistance. This phenomenon is particularly prevalent in triple-negative breast cancer (65). Methods based on a 2-minute half-life ^15^O are effective for perfusion assessment (66), however the use of cyclotrons limits its clinical application. Neree Payan et al. used a first-pass scanning approach with a 2-minute dynamic acquisition centered on the chest immediately after injection of ^18^F-FDG, followed by a static acquisition 90 min later, which correlated well with ^15^O-water measurements of tumor perfusion. In patients with lymph node involvement, primary tumors exhibited high perfusion and K_1_ values (67), which may explain the extensive angiogenesis that may promote cancer metastasis.

### 4.4 Pancreatic carcinoma

Pancreatic cancer is a malignant tumor the primarily originates from pancreatic ductal epithelial and follicular cells, with high degree of malignancy and insidious onset. Among its various subtypes, pancreatic ductal adenocarcinoma cancer (PDAC) is the most common type, accounting for about 90% of the cases (68). Intraductal papillary mucinous neoplasm (IPMN) is the precursor of malignant transformation of PDAC (69). Although many IPMNs are benign, differentiating between these and those with malignant potential is crucial. Matthias Lang et al. demonstrated that dPET parameters (TTP, K_1_, K_2_, K_3_, K_4_) can effectively distinguish between high-grade and low-grade IPMNs. With 80% sensitivity and specificity, this approach offers a promising tool for predicting IPMN subtypes and potentially avoiding unnecessary surgery for non-malignant lesions (70). In terms of staging and prognosis of PDCA, the dual imaging with ^68^Ga-FAPI and ^18^F-FDG allowed precise stratification of postoperative patients, with 18.4% and 10.2% of patients experiencing a change in staging, respectively, but ^68^Ga-FAPI had a lower specificity in identifying primary lesions (71) that was mainly attributed to the overlapping uptake intensity of primary pancreatic tumors and tumor-induced obstructive pancreatitis of the pancreatic parenchyma. Unfortunately, dynamic ^68^Ga-FAPI-04 PET/CT did not effectively differentiate between PDAC and distal obstructive pancreatitis based on K_i_ values (72). However, dual time-point imaging with a 3-hour delay successfully distinguished between tumor and inflammation-induced fibrosis (72).

Currently, there are limited studies on the dynamics of PET/CT in pancreatic cancer, especially with the low sensitivity or specificity of ^18^F-FDG (73), the development of new imaging agents is imperative, and the use of ^68^Ga-labeled trimerized αvβ6 integrin-selective nonpeptide ^68^Ga-Trivehexin has been shown to be superior in PDAC metastases, especially in cancers with low metabolic conversion and/or high stromal ratios, such as liver (74), brain tissue and retroperitoneum (75). In addition, researchers are actively seeking innovative strategies to enhance pancreatic cancer treatment outcomes. A primary focus of targeted therapy involves identifying new targets through the administration of short-lived, highly radioactive radionuclides. In the study by Tadashi Watabe et al., dPET was employed to evaluate ^64^Cu and ^225^Ac to label mice with human pancreatic cancer xenografts using a FAP inhibitor (FAPI) and found that it significantly inhibited tumor growth (76), while Yuwei Liu et al. discovered that administration of ^177^Lu-FAPI-46 and ^225^Ac-FAPI-46 to PANC-1 xenograft mice decreased the tumor growth. However, ^177^Lu-FAPI-46 exhibited a less intense but more prolonged therapeutic effect compared with ^225^Ac-FAPI-46 (77). These studies suggest that β-therapy and α-therapy targeting FAP may offer effective treatment of pancreatic cancer, but it is currently in the clinical translational phase and further investigations using dPET are needed to determine the best mix of rapid FAP kinetics and physical radionuclide decay. There is a compelling need to develop combinatorial treatment strategies focused on tumor cell elimination.

### 4.5 Prostate neoplasm

Current statistics indicate that prostate neoplasm has surpassed lung cancer as the most common tumor in men worldwide and is the leading cause of cancer deaths in men (57). Since 2020, when ^68^Ga-PSMA-11 was approved by the FDA (78), prostate-specific membrane antigen (PSMA)-targeted PET imaging of prostate cancer employing ^68^Ga-labelled complexes is increasingly being adopted in routine clinical treatment worldwide. Studies have shown that the kinetic behavior of ^68^Ga-PSMA-11 varies across different organs and lesions. The irreversible two-tissue compartmental (2T4k) model is more suitable for describing the kinetics in pathological lesions, while the reversible two-tissue compartmental (2T4k) model better fits normal tissue kinetics. In terms of kinetic metrics, the K_3_ value effectively distinguished between abnormal lesions and healthy organs with increased absorption of ^68^Ga-PSMA-11, and is expected to gain broader clinical application as an imaging biomarker for differentiating pathological and non-specific ^68^Ga-PSMA-11 uptake in prostate cancer patients (79). Currently, delayed imaging with ^68^Ga-PSMA PET/CT can improve the identification of non-specific PSMA ingestion in patients with prostate cancer (80). In addition, K_i_ images collected using whole-body PET/CT scanning for 60 min are less noisy and have sharper lesions than SUV images, making them ideal for early detection of small lesions (81). The lengthy 60-min scan time can lead to significant bladder activity, hindering the detection of small lesions like local recurrences or lymph node metastases near the bladder. A combination of a 6-min early dynamic imaging and static imaging protocol (91), as proposed by Jun Wen et al., effectively reduces bladder interference and shortens the PET/CT acquisition time to 45 min. While the improvement in image quality is modest, it enhances patient comfort (82).

The longer half-life of ^18^F compared to ^68^Ga offers great convenience in terms of transportation and can be used in delayed imaging protocols. Evidence indicates that ^18^F-DCFPyL and ^68^Ga-PSMA-11 exhibit similar biodistribution patterns including the bladder uptake, and that ^18^F-DCFPyL has a higher overall detection rate although it did not show a higher detection rate in terms of localized foci (83), this may be due to the better spatial resolution of the images provided by ^18^F. A 22-min dynamic ^18^F-DCFPyL PET scan effectively detected dominant intraprostatic lesions. Kinetic parameters K_i_ and K_4_ derived from this scan accurately differentiated between tumor and benign tissue (84). These investigations suggest that ^18^F-DCFPyL is the most favorable substitute for ^68^Ga-labeled drugs. Moreover, the transition from ^68^Ga-labeled PSMA-targeted compounds to ^18^F-labeled PSMA-targeted compounds is expected to gain broader clinical acceptance.

### 4.6 Head and neck tumors

Hypoxia is a common feature of head and neck cancer and a predictor of poor prognosis following radiotherapy (85). ^18^F-Fluoromisonidazole (FMISO) has been extensively investigated as a radiotracer for hypoxic PET with good performance (86). Tumor-to-Blood Ratio and Tumor-to-Muscle Ratios are measurements obtained from static FMISO scans and are frequently employed as substitute biomarkers for tumor hypoxia, depending on the specific moment of image capture. Furthermore, tumor-to-blood ratio and tumor-to-muscle ratios values are influenced by the distribution of FMISO within and among tumors. Consequently, this method may inaccurately assess the presence and severity of hypoxia. Milan Grkovski et al. performed a 30-min single-bed dynamic imaging of 120 patients with head and neck cancer, and found that ^18^F-FMISO dPET provided data required for parametric mapping of tumor hypoxia, perfusion, and radiotracer distribution volumes, and K_3_ was identified as a direct biomarker of hypoxia-mediated ^18^F-FMISO accumulation, and K_3_ was not dependent on the volume of distribution and time of acquisition with FMISO (87).

Tumor perfusion is a critical factor influencing drug delivery. Changes in perfusion can serve as a prognostic indicator for the effectiveness of both conventional and targeted therapies, such as anti-angiogenic treatments (88). Studies have reported that tumor perfusion and hypoxia can help to predict response to treatment (89). The 20-min truncated ^18^F-FMISO dPET scans, i.e., by the kinetic parameters K_1_ and K_3_ reacting to tumor hypoxia and perfusion can identify alternative markers of hypoxia and perfusion. Currently, two hypotheses have been proposed for the relationship between K_1_ and K_3_, one is a negative correlation, whereby hypoxia occurs in a hypoperfused tumor volume with a disorganized and dysfunctional microvascular structure. It is hypothesized that the increased separation between tumor cells and functional blood vessels results in diminished oxygen delivery to distal cells, causing diffusion-limited hypoxia. The other is a positive correlation, which implies that areas with good perfusion may experience hypoxia (89). This may reflect the various forms of hypoxia (patchy, banded, and mixed) described in head and neck tumors by Ljungkvist et al, who reported that hypoxia and perfusion can simultaneously occur in very close proximity under the microscope (90). Notably, hypoxia may promote angiogenesis, hyperperfusion and perfusion heterogeneity.

## 5 Conclusions

Radiotracer-based kinetic modeling and dynamic imaging provide data that cannot be obtained by conventional static PET/CT, and have been successfully applied in various malignant tumors. They provide kinetic scanning parameters are of superior value for diagnosis, differential diagnosis, and assessment of treatment response and prognosis in these diseases than the visual and semiquantitative analysis of conventional static imaging. The practical application of kinetic imaging is hindered by issues related to data volume, scan duration, and the sophistication required for data analysis. With the advent of state-of-the-art PET/CT scanners, these problems can be ameliorated with the optimization of existing clinical applications of whole-body PET/CT, such as sequential dPET imaging of all relevant organ structures to map the pharmacokinetic behavior of (new) tracers. Moreover, exploring the potential of artificial intelligence in image analysis could lead to advanced multiparametric methods and uncover new avenues for research.
